# Generation in yeast of recombinant virus-like particles of porcine circovirus type 2 capsid protein and their use for a serologic assay and development of monoclonal antibodies

**DOI:** 10.1186/s12896-014-0100-1

**Published:** 2014-12-09

**Authors:** Juozas Nainys, Rita Lasickiene, Rasa Petraityte-Burneikiene, Jonas Dabrisius, Raimundas Lelesius, Vilimas Sereika, Aurelija Zvirbliene, Kestutis Sasnauskas, Alma Gedvilaite

**Affiliations:** Institute of Biotechnology, Vilnius University, Graiciuno 8, LT-02241 Vilnius, Lithuania; Institute of Microbiology and Virology, Veterinary Faculty of Veterinary Academy, Lithuanian University of Health Sciences, Tilzes 18, LT-47181 Kaunas, Lithuania

**Keywords:** Virus-like particles, Porcine circovirus 2, Monoclonal antibodies

## Abstract

**Background:**

Porcine circovirus type 2 (PCV2) is considered to be an important emerging pathogen associated with a number of different syndromes and diseases in pigs known as PCV2-associated diseases. It has been responsible for significant mortality among pigs and remains a serious economic problem to the swine industry worldwide leading to significant negative impacts on profitability of pork production.

**Results:**

In this study we have demonstrated that PCV2 capsid (Cap) protein based virus-like particles (VLPs) were efficiently produced in yeast *S. cerevisiae* and induced production of monoclonal antibodies (MAbs) reactive with virus-infected cells. Moreover, PCV2 Cap VLPs served as a highly specific recombinant antigen for the development of an indirect IgG PCV2 Cap VLP-based ELISA for the detection of virus-specific IgG antibodies in swine sera. Four hundred-nine serum samples collected from pigs in Lithuania were tested for PCV2-specific IgG to determine the sensitivity and specificity of the newly developed ELISA in parallel using a commercial SERELISA test as a gold standard. From 409 tested serum samples, 297 samples were positive by both assays. Thirty-nine sera from 112 serum samples were determined as negative by SERELISA but were found to be positive both in the newly developed indirect IgG PCV2 Cap VLP-based ELISA and the PCR test.

**Conclusions:**

We have demonstrated that *S. cerevisiae* expression system is an alternative to insect/baculovirus expression system for production of homogenous in size and shape PCV2 Cap protein-based VLPs similar to native virions. Yeast expression system tolerated native virus genes encoding PCV2 Cap protein variants as well as the codon-optimized gene. Moreover, yeast-derived PCV2 Cap VLPs were capable to induce the generation of PCV2-specific MAbs that did not show any cross-reactivity with PCV1-infected cells. The high sensitivity and specificity of the indirect IgG PCV2 Cap VLP-based ELISA clearly suggested that this assay is potentially useful diagnostic tool for screening PCV2–suspected samples.

## Background

Circoviruses belong to the Circoviridae family and infect a variety of animal and plant species. They are named after their circular single-stranded DNA (ssDNA) genome and represent the smallest DNA viruses infecting mammals. Porcine circovirus 2 (PCV2) is found all over the world in the domestic pigs and the wild boars which seldom display the clinical condition. PCV2 serological prevalence in the wild boars is almost as high as in domestic pigs [1]. PCV2 is considered to be an important emerging pathogen associated with a number of different syndromes and diseases in pigs known as PCV2-associated diseases (PCVAD). Overall, PCVAD includes clinical syndromes and diseases such as postweaning multisystemic wasting syndrome (PMWS), porcine dermatitis and nephropathy syndrome (PDNS), porcine reproductive disorders, respiratory disease complex, granulomatous enteritis, congenital tremor, necrotizing tracheitis and lymphadenitis and exudative epidermitis [2-6]. PCV2 has been responsible for significant mortality among pigs and remains a serious economic problem to the swine industry worldwide leading to significant negative impacts on profitability of pork production [2]. The ubiquitous nature of the virus and the retrospective evidence of this infection long before disease appearance provided the reason for debate about the real capabilities of this virus in its association with the disease. How the virus causes disease is still not fully understood. Infection with PCV2 in combination with still unknown co-factors can cause immunopathological disorders such as PMWS, or induce protective immunity. It might be that PCV2-induced diseases are the result of PCV2-caused immune disorder. It was demonstrated that pigs with PMWS display a marked depletion of T- and B-lymphocytes, and their lymphoid organs are infiltrated by histiocytes and multinucleated giant cells and might cause deficiency of total and neutralizing antibody response [7,8]. The interaction of PCV2 with the innate immune system have been mostly studied *in vitro* looking for the effects of the virus on dendritic cells (DCs), macrophages and peripheral blood mononuclear cells [7]. It was shown that PCV2 infection of plasmacytoid DCs impairs the induction of interferon-α and tumor necrosis factor-α, which silences responsiveness to pathogen-associated recognition signals by compromising immune defense development against other pathogens. As a result this may lead to a host susceptibility to secondary infections which actually are responsible for developing of PCVAD [9].

PCV2 is a non-enveloped virus with circular single stranded DNA molecule of approximately 1,767 bases packed in icosahedral 12–23 nm diameter virion particle. Virus genome sequences with nucleotide diversity cut-off of 3.5% were divided into four genotypic groups [1]. The two major groups designated as PCV2a, PCV2b are found worldwide and have been demonstrated to be virulent enough to trigger PCVAD [1,10,11]. The two more genotype types - PCV2c reported from Denmark in the 1980s and PCV2d found in China are not distributed so widely [12,13].

The PCV2 genome contains two major open reading frames (ORFs). The replicase (Rep and Rep’) proteins responsible for initiation of viral replication are encoded by ORF1 located on the viral plus strand. A sole structural protein of the viral coat encoded by ORF2 on complementary strand is the unique structural capsid (Cap) protein [14]. Cap protein is the major immunogenic protein containing specific epitopes of PVC2 [15]. The Cap protein reacts strongly with the serum of PCV2-infected pigs and therefore, it is a major candidate antigen for the development of serological diagnostic methods.

There were multiple attempts to produce recombinant Cap protein in different expression systems including *E. coli*, *Lactococcus lactis, Pichia pastoris*, baculovirus or pseudorabies virus expression systems [15-21]. In most cases the expression of ORF2 gene of PCV2 was achieved by optimizing codon composition, removing N terminal part of the protein rich of arginine residues or by adding different tags useful for purification. These approaches have been shown to be successful to yield truncated or full length monomeric Cap protein. Expression of PCV2 Cap in insect cells resulted in recombinant protein self-assembled into virus-like particles (VLPs) that were structurally and antigenically indistinguishable from regular PCV2 capsids [15]. Recombinant VLPs due to their repetitive surface epitopes, the virus-like structure and the capability to induce humoral and/or CTL responses are more suitable for the development of immunogenic vaccines and sensitive diagnostic tests as compared to monomeric viral antigens [22]. At present, few baculovirus-derived PCV2a subunit vaccines are available [23]. PCV2 Cap VPLs produced in baculovirus expression systems were shown to be a suitable antigen for detection of PCV2 infection by the indirect IgG PCV2 ELISA [24,25]. However, the possibilities to employ cheaper antigens for diagnostic purposes are considered mostly by using monomerus PCV2 Cap protein [18,25-27].

In the current study we demonstrate that PCV2b-derived Cap VLPs can be efficiently produced in yeast *Sacharomyces cerevisiae* and are excellent antigens for generation of monoclonal antibodies (MAbs) reactive with virus-infected cells. Moreover, we demonstrate that yeast-derived recombinant PCV2 Cap VLPs represent a promising antigen for a highly sensitive and specific immunoassay for serologic diagnosis of PCV2 infection.

## Results

### Production of PCV2 VLPs in yeast

PCV2-positive samples collected between 2009 and 2013 from domestic pigs from 9 regions of Lithuania were used for the amplification of full-length PCV2 genomic DNA with overlapping primers. DNA sequence alignment of the 45 cloned and sequenced PCV2 genomes circulating in Lithuania revealed six slightly different genome variants (GenBank: KJ128269, KJ128270, KJ128271, KJ128272, KJ128273, KJ128274). All six PCV2 genome variants belonged to genotype PCV2b according to the previously proposed criteria [10,11].

Three open reading frame 2 (ORF2) variants amplified by PCR from native PCV2 genomes: variant 622 (GenBank: KJ128269), variant 521 (GenBank: KJ128273) and variant 113 (GenBank: KJ128274) as well as ORF2 of the most representative PCV2-Cap gene variant 622 which was codon-optimized by *S. cerevisiae* codon usage (GenBank: KJ128275) were cloned into pFX7 expression vector [28] and used for the expression in yeast *S. cerevisiae*.

**Figure 1:**
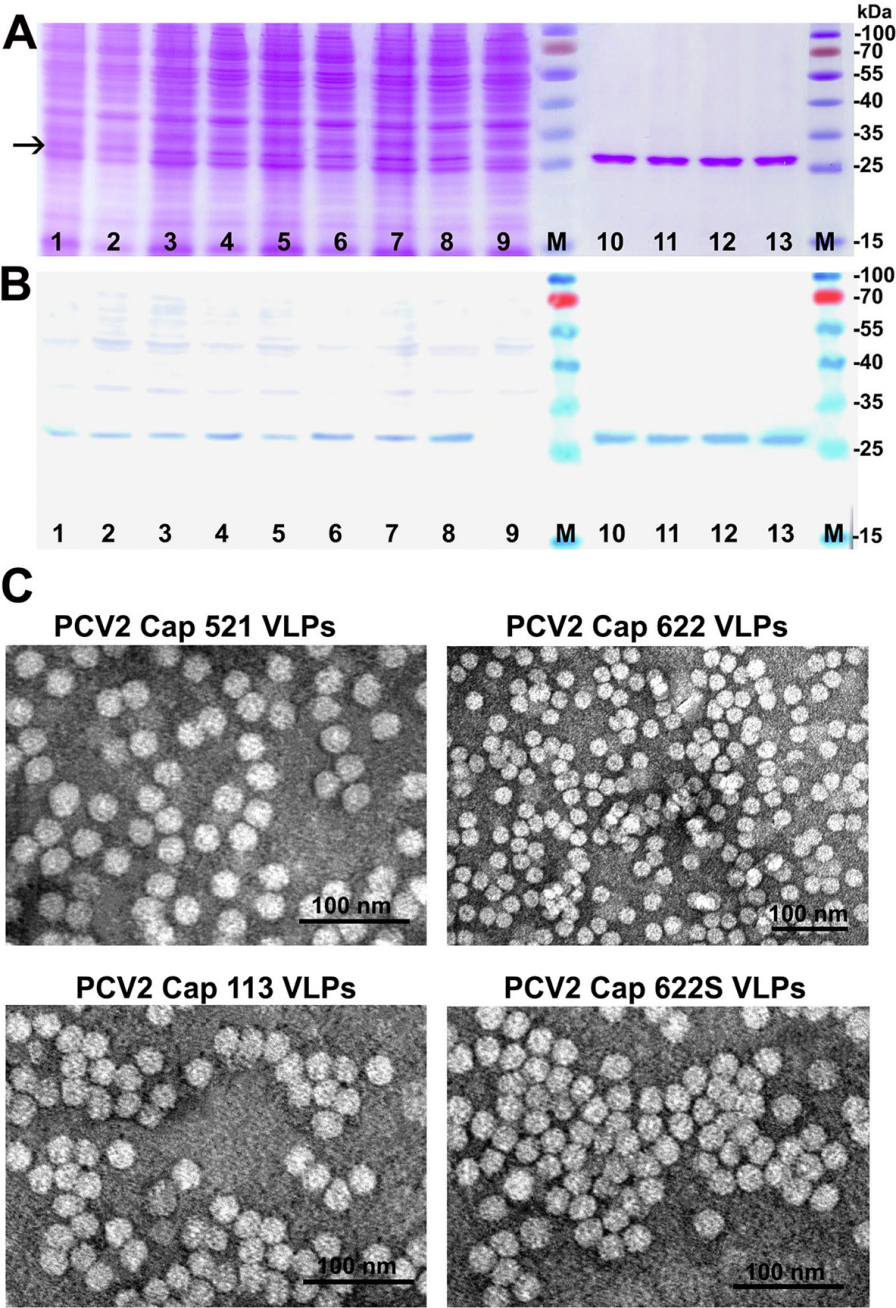
**Analysis of recombinant PCV2 Cap protein-derived VLPs produced in yeast. (A)** Coomassie blue-stained SDS-PAGE; **(B)** Western blot with pig serum. **(A,B)**: In lanes: 1 - whole crude lysate of yeast transformed with pFX7-PCV2Cap-521 plasmid, 2 - the soluble fraction of the lysate of yeast transformed with pFX7-PCV2Cap-521 plasmid; 3 - whole crude lysate of yeast transformed with pFX7- pFX7-PCV2Cap-113 plasmid; 4 - the soluble fraction of the lysate of yeast transformed with pFX7-PCV2Cap-113 plasmid; 5 - whole crude lysate of yeast transformed with pFX7-PCV2Cap-622 plasmid; 6 - the soluble fraction of the lysate of yeast transformed with pFX7-PCV2Cap-622 plasmid; 7 - whole crude lysate of yeast transformed with pFX7-PCV2Cap-622S plasmid; 8 - the soluble fraction of the lysate of yeast transformed with pFX7-PCV2Cap-622S plasmid; 9 - whole crude lysate of yeast transformed with pFX7 plasmid, M- pre-stained protein weight marker (Thermo Fisher Scientific Baltics); 10 - Cap521 protein VLPs purified by density gradient centrifugation; 11 - Cap113 protein VLPs purified by density gradient centrifugation; 12 – Cap622 protein VLPs purified by density gradient centrifugation; 13 - Cap622S protein VLPs (Cap622 protein VLPs generated using synthetic codon-optimized gene) purified by density gradient centrifugation. **(C)**: Electron microscopy pictures of VLPs formed by PCV2-Cap521, protein PCV2-Cap113, protein PCV2-Cap622 and protein PCV2-Cap622S protein stained with 2% aqueous uranyl acetate solution and examined by Morgagni 268 electron microscope.

The expression of four cloned genes was detected in SDS-PAGE and confirmed by Western blot using pig serum (Figure 1A,B). All recombinant PCV2-Cap proteins were found in the soluble fraction of yeast cell lysate which allowed their efficient purification (Figure 1A, B, lanes 2, 4, 6, 8). All four variants of PCV2-Cap proteins generated in yeast were purified by density gradient centrifugation and examined using electron microscope (EM). Despite the slight differences in expression levels of PCV2-Cap gene variants, all four purified Cap proteins were assembling into regular homogenous in size VLPs about 17–22 nm in diameter similar to native PCV2 virions (Figure 1C). PCV-Cap VLPs encoded by the most representative PCV2-Cap gene variant 622 were used for generation of MAbs and development of an indirect ELISA for detection of PCV2-specific IgG.

### Generation and characterization of monoclonal antibodies against PCV2b-Cap VLPs

To generate the MAbs, mice were immunized with recombinant PCV2 Cap 622 VLPs and tested for the presence of Cap-specific serum antibodies. Spleen cells of a mouse showing the highest titer of Cap-specific antibodies were used to generate hybridomas. After fusion, all hybrid clones were screened for the reactivity with PCV2 Cap VLPs and specific ones were subjected to isotyping and recloning. After these procedures, three stable hybridoma cell lines producing PCV2 Cap-specific MAbs of IgG isotype were obtained. Two of them (clones #14G10 and #7C4) produced MAbs of IgG2a subtype and one clone (#24C7) of IgG1 subtype. The specificity of all three MAbs was proven by ELISA using all three variants of yeast-expressed Cap proteins: PCV2 Cap521 protein, PCV2 Cap113 protein, and PCV2 Cap622 protein. No reactivity of the MAbs with Hantaan (Fojnica) and Tioman nucleocapsid proteins [29,30] used as a negative control was observed, which confirmed the specificity of these MAbs to the PCV2 Cap protein (data not shown).

Western blot analysis of MAb specificity using the lysate of yeast expressing PCV2 Cap protein revealed that only one MAb (clone #14G10) recognized SDS-denaturated PCV2 Cap protein (data not shown). Meanwhile, the MAbs #7C4 and #24C7 did not react with PCV2 Cap protein in Western blot thus indicating that they are directed against conformational epitopes (data not shown).

**Figure 2:**
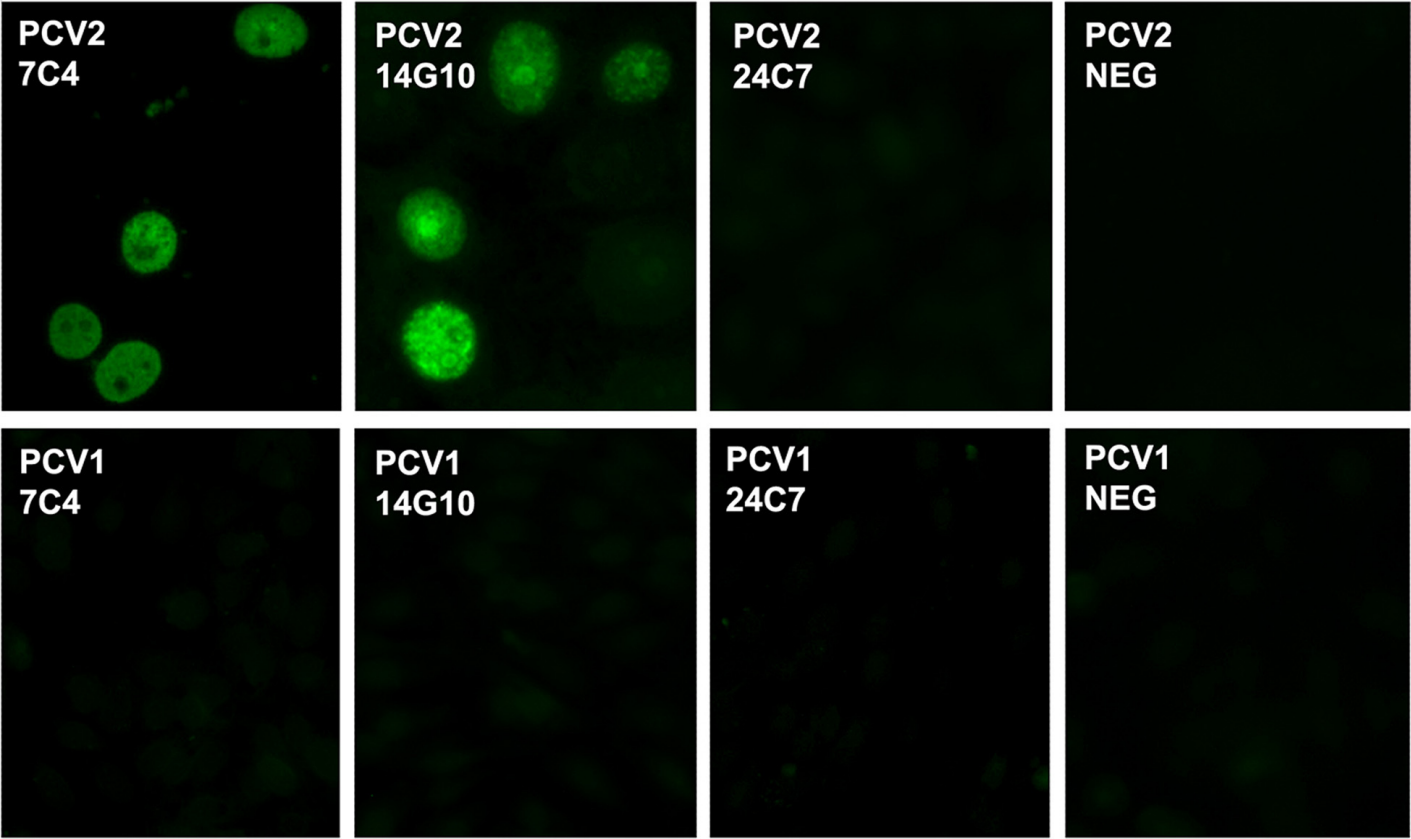
**Indirect immunofluorescence staining of PCV2 infected swine testicle (PT-1) cells and PCV1-infected PK cells with MAbs #7C4, #14G10 and #24C7.** NEG - MAb #4 F11 directed against parainfluenza type 3 N protein [41].

To evaluate the diagnostic potential of the generated MAbs, their reactivity with PCV2-infected (virus strain T-657) swine testicle cells (PT-1) and PCV1-infected porcine kidney (PK) cells was tested by an indirect immunofluorescence assay (IFA) using commercially available slides (VMRD, Inc, Pullman, WA, USA). The MAbs #7C4 and #14G10 were reactive with PCV2-infected swine testicular cells but did not recognize PCV1-infected PK cells thus confirming their specificity to PCV2 Cap protein (Figure 2). Meanwhile, the MAb #24C7 was not reactive in both IFA tests.

### Development of an indirect ELISA for detection of PCV2 Cap-specific IgG

**Figure 3:**
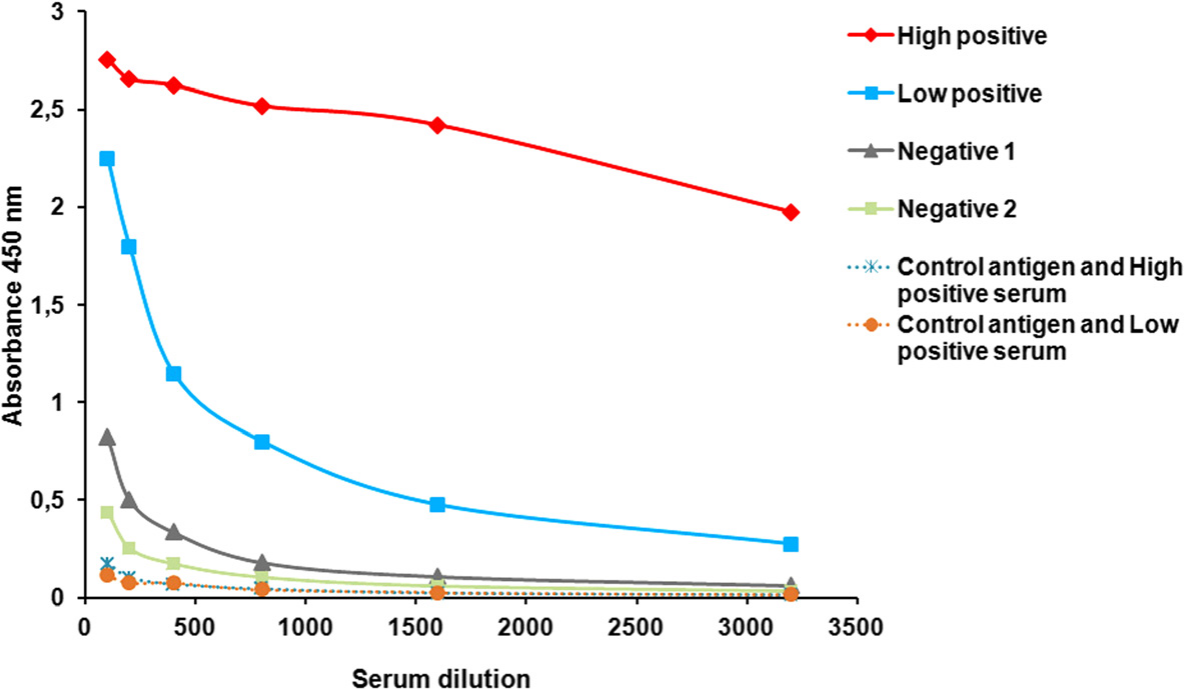
**Titration curves of swine serum samples.** Either positive or negative for PCV2 antibodies swine serum samples were titrated by the newly developed indirect IgG PCV2 Cap VLP-based ELISA using recombinant PCV2 Cap VLPs at a concentration of 0.5 μg/ml (solid lines) and control antigen (HaPyV-VP1) at a concentration of 2 μg/ml (dotted line). Sera were tested in serial duplicate twofold dilutions ranging from 1:100 to 1:3200. The results are expressed as the OD_450_ and correspond to the average of the values obtained in at least two different analyses.

**Figure 4:**
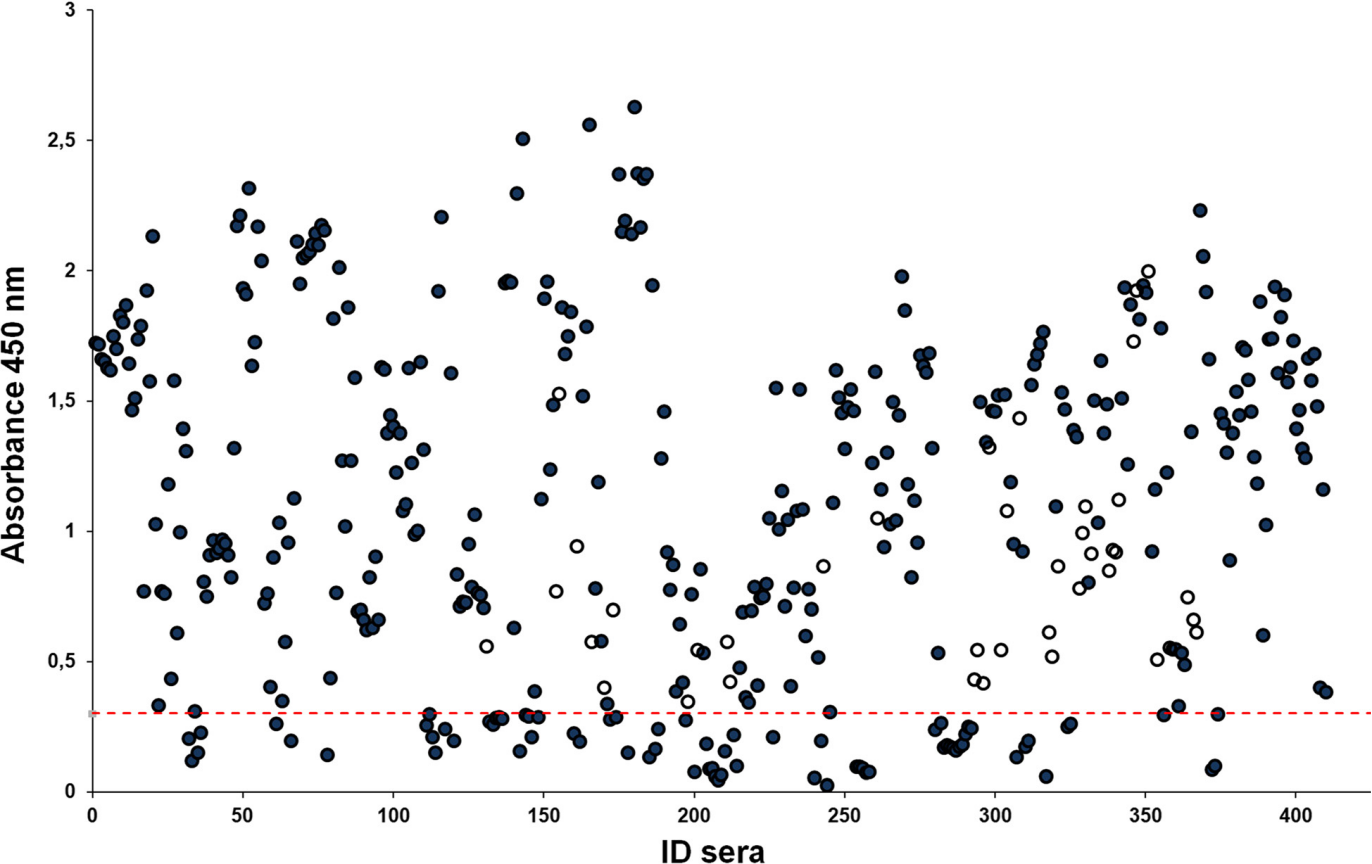
**Optical density results of serum samples in the newly developed indirect IgG PCV2 Cap VLP-based ELISA.** Four hundred and nine serum samples previously characterized by the commercial SERELISA test were analyzed by the newly developed indirect PCV2-Cap VLP IgG ELISA. The absorbance at 450 nm obtained for the serum samples are shown. The dotted line indicates the assay cut-off, which was established to be 0.3. for the indirect PCV2 Cap VLP IgG ELISA. Opened circles represent discrepant results, filled circles indicate matching results with commercial SERELISA test. ID sera, serum sample number.

As a first step in the development of PCV2-Cap VLP-based ELISA, checkerboard titrations were performed to determine the optimal concentration of the Cap antigen and the test serum. To optimize plate coating, the recombinant PCV2-Cap 622 protein was immobilized on ELISA plates at five different concentrations: 2; 1; 0.5; 0.25; 0.125 μg/mL. The optimal antigen concentration for plate coating as determined by a checkerboard titration was 0.5 μg/mL. To determine the optimal serum sample dilution, serum samples identified by the commercial SERELISA test (Synbiotics, Lyon, France) as high positive, low positive and negative were diluted in serial two fold dilutions ranging from 1:100 to 1:3200. The OD450 values obtained in an indirect ELISA were plotted against the dilutions (Figure 3). High OD readings with positive control serum and low background signals with negative sera were obtained at serum dilution 1:800. The absorbance values declined at serum dilution 1:1600. As the commercial SERELISA test uses serum dilution 1:1000 and the differences between OD values at serum dilutions 1:800 and 1:1000 were insignificant in our newly developed ELISA, for convenience the appropriate dilution (1:1000) was selected for the indirect IgG PCV2 Cap-based ELISA. Control antigen – yeast expressed Hamster polyomavirus (HaPyV) VP1 protein VLPs [28] was included in the test to determine the specificity of the assay. The positive samples revealed significantly higher OD values (0.9–2.5) with PCV2 Cap protein as compared to the control HaPyV-VP1 antigen (0.05–0.1) (Figure 4, Dotted lines).

The cut-off value was calculated as the mean OD450 value of 42 serum specimens negative by SERELISA plus 3 standard deviations (mean +3SD). This calculation gives 99% confidence. The mean OD450 value and SD were 0.146 and 0.053, respectively. Therefore, the positive threshold determined for the newly developed indirect IgG PCV2 Cap VLP-based ELISA was 0.3. A serum sample was considered positive when its OD450 value was greater than the positive threshold (OD450 > 0.3).

Four hundred-nine serum samples were tested to determine the sensitivity and specificity of the newly developed ELISA. The sensitivity and specificity were calculated for the indirect IgG PCV2-Cap VLP-based ELISA using SERELISA test as a gold standard. All serum samples were tested in parallel with a commercial SERELISA kit. From 409 serum samples tested, 297 samples were positive by both assays. Thus, the calculated sensitivity of the new ELISA test was 100% (297/297).

One hundred-twelve serum samples negative by SERELISA were tested to determine the specificity of the indirect IgG PCV2-Cap VLP-based ELISA. Seventy-three sera were below and 39 serum samples were above the cut-off by indirect IgG PCV2-Cap VLP-based ELISA (Figure 4, Table 1). Indirect IgG PCV2-Cap VLP-based ELISA identified these samples as positive (Figure 4, samples labeled with opened circles), although in the commercial SERELISA test these samples were found to be negative. Discrepant serum samples were re-tested by PCR. Overall, all samples of the suspected PCV2 cases were PCV2 DNA positive. Thus, samples positive by the newly developed indirect IgG PCV2-Cap VLP-based ELISA were confirmed by PCR but missed by commercial SERELISA IgG test. All serum samples were tested in duplicate and the correlation coefficient (r-value) between two measurements was calculated. The r value was shown to be 0.972, which indicates very high reproducibility between tests.

**Table 1:** **Comparative evaluation of the indirect IgG PCV2 Cap VLP-based ELISA with commercial SERELISA kit for detection of anti-PCV2 antibodies**

**Indirect IgG ELISA**	**Commercial SERELISA test**		
	**Positive**	**Negative**	**Total**
Positive	297	39	**336**
Negative	0	73	**73**
**Total**	**297**	**112**	

The values represent the numbers of sera tested.

These results indicate that the newly developed indirect IgG PCV2-Cap VLP-based ELISA had higher sensitivity as compared to the commercial SERELISA test, which failed to detect some PCV2-positive samples as positives.

## Discussion

Virus-like particles present viral antigens in a more authentic conformation than monomeric structural proteins. Therefore, recombinant VLPs are more preferable for vaccine or diagnostic purposes [22]. VLPs mimic the structure of authentic virus particles they are derived from and are easily recognized by the immune system and able to stimulate both B-cell and T-cell immune responses. Multiple expression systems such as *E. coli, L. lactis, P. pastoris* allowed generation of monomeric PCV2 Cap protein but not VLPs [16-20]. Different modifications of the gene encoding PCV2 protein were also necessary for generation of recombinant PCV2 Cap protein including removal of the N terminal part with nuclear localization signal and fusing it with different tags or yeast signal peptide as well as the optimization of codon usage according to the host [16-21]. Even for the investigation of the structure of PCV2 Cap-derived VLPs, both the full length and an N-terminally truncated monomeric Cap protein was purified from *E.coli* and reassembled into VLPs *in vitro* [31]. On the other hand, baculovirus/insect cell expression system supported PCV2-Cap protein self-assembly into VLPs [14,15]. Yeast *Saccharomyces cerevisiae* might provide an alternative expression system for PCV2 Cap protein production as there are several reports which confirm that yeast can support VLP auto-assembly of viral proteins [32-34]. In a Bucarey’s study, the failure of PCV2 Cap protein production in *S.cerevisiae* using native PCV2-Cap coding sequence was reported [35]. It was demonstrated that only codon optimized gene allowed generation of PCV2 Cap VLPs in yeast. Moreover, in Bucarey’s study generated VLPs were not homogenous in size and shape [35]. This is opposite to our results presented in the current study (Figure 1). We demonstrate that PCV2b-Cap VLPs were efficiently produced in yeast *S.cerevisiae* using native virus ORF2 versions of Cap-encoding genes as well as the codon-optimized gene. We purified three slightly different PCV2 Cap proteins variants encoded by native Cap-encoding sequences and confirmed that they all formed homogenous in size and shape VLPs similar to native virions or VLPs produced in baculovirus expression system [14].

Yeast-produced PCV2 Cap VLPs were highly immunogenic and induced PCV2-specific immune response in mice. We generated three MAbs with different capabilities to recognize both the recombinant PCV2 Cap protein and virus-infected cells. The MAb #14G10 demonstrated exclusive and very specific reactivity with linear PCV2 Cap protein epitope in Western blot, ELISA and IFA implying the localization of this epitope on the surface of VLPs. The MAb #24C7 was non-reactive with virus-infected cells and did not recognize SDS-denaturated PCV2 Cap protein, which suggests that its epitope is accessible only in native PCV2 Cap VLPs. The MAb #7C4 also recognized conformational PCV2 epitope but it was accessible on virus-infected cells as demonstrated in IFA (Figure 2). All three MAbs were highly specific and did not show any cross-reactivity with PCV1 Cap protein in all assays despite a significant homology between PCV2 and PCV1 (a non-pathogenic virus) in terms of amino-acid sequences. Therefore, the newly generated MAb #7C4 and especially the MAb #14G10 could be used as highly specific reagents for PCV2 detection by IFA or monoclonal antibody-based blocking ELISA.

The Cap protein is a major candidate antigen for the development of serologic diagnostic methods as it is the major immunogenic protein containing specific epitopes of PVC2 [15]. In the previous studies recombinant PCV2 Cap protein expressed in either prokaryotes or eukaryotes was used in an indirect ELISA as coating antigen. It was shown that PCV2 Cap VLPs or Cap protein expressed in insect cells as well as Cap protein expressed in *E. coli* showed high immunoreactivity with serum antibodies [18,24-26]. The diagnostic sensitivity, specificity, and accuracy of ELISAs performed with PCV2 Cap VLPs and PCV2 were similar (>90%) to IFA [24]. However the sensitivity of ELISA’s developed using *E. coli*-expressed recombinant proteins were lower. This is likely because natural virus particles are perfect in structure whereas recombinant *E. coli*-expressed proteins are not always refolded completely. Sun and colleagues developed protein-based ELISA for PCV2 specific diagnosis using *E. coli*-expressed antigenic domain of PCV2 Cap protein [36]. The developed assay failed to detect some PCV2-positive serum samples identified by IFA. The specificity was lower also in a monoclonal antibody-based blocking ELISA developed by Huang and colleagues [37]. Its sensitivity and specificity were determined to be 98.8% and 88.5%, respectively. Higher sensitivity was achieved in a quantitative immunofluorescence assay (QIFA) using the recombinant PCV2 nucleocapsid protein expressed in Vero cells by a lentivirus system, but the specificity was lower when compared to ELISA [38]. In the current study, the newly developed indirect IgG PCV2-Cap VLP-based ELISA proved to be a highly sensitive and specific tool for the serologic diagnosis of PCV2 infection and was even more sensitive than the commercial SERELISA test. It might be that not only VLP structure of yeast-expressed Cap protein influenced the higher sensitivity of the newly developed indirect IgG PCV2-Cap VLP-based ELISA. Based on the GenBank database and several studies published in different countries a shift on genotype prevalence from PCV2a to b have been observed [1]. Our analysis of PCV2 genotype spread in Lithuania pig farms in the samples collected between 2009 and 2013 confirmed that all isolated virus genomes belonged to the PCV2b genotype. Most of commercial diagnostic tests are based on PCV2a genotype-derived Cap proteins. Our preliminary data showed that some PCV2-negative serum samples identified by a commercial SERELISA test were negative in an indirect IgG Cap VLP-based ELISA when PCV2a genotype-derived Cap VPLs were used but were positive when PCV2b genotype-derived Cap VLPs were used as a coating antigen. However to prove the hypothesis more serological assays should be performed.

## Conclusions

Recombinant VLPs are more preferable for vaccine development or diagnostic purposes as they present viral antigens in a proper conformation. In this study we have demonstrated that *S.cerevisiae* expression system is an alternative to insect/baculovirus expression system for production of homogenous in size and shape PCV2 Cap protein-based VLPs similar to native virions. Yeast cultures are easy to maintain, which makes them faster and less expensive to use than other eukaryotic expression systems. We showed that yeast expression system tolerated native virus genes encoding PCV2 Cap protein variants as well as the codon-optimized gene. Yeast-derived PCV2 Cap VLPs were capable to induce the generation of PCV2-specific MAbs that did not show any cross-reactivity with PCV1-infected cells. Moreover, the employment of PCV2 Cap VLPs as an antigen in the indirect IgG PCV2 Cap VLP-based ELISA demonstrated that this newly developed PCV2 diagnostic test was more sensitive and specific than the commercial SERELISA test and is potentially useful as a diagnostic assay in screening PCV2-suspected swine samples.

## Methods

### Generation of yeast expression plasmids

All DNA manipulations were carried out according to standard procedures [39]. Enzymes and kits for DNA manipulations were purchased from UAB “Thermo Fisher Scientific Baltics” Vilnius, Lithuania). Recombinants were screened in *E. coli* DH5α cells.

PCV2 genome sequences were amplified using Phusion High-Fidelity DNA Polymerase (Thermo Fisher Scientific Baltics) from PCV2-positive samples of domestic pigs collected between 2008 and 2013 from 9 regions of Lithuania, sequenced with overlapping primers and aligned. PCV2 genome sequences with nucleotide variations were submitted to GenBank (GenBank: KJ128269, KJ128270, KJ128271, KJ128272, KJ128273, KJ128274). DNA sequences encoding three different variants of PCV2 capsid protein sequences - 622 (GenBank: KJ128269), 521 (GenBank: KJ128273) and 113 (GenBank: KJ128274) - were PCR amplified from DNA isolated from porcine serum or tissue samples collected in Lithuanian pig farms. Collection of samples was carried out in accordance with institutional guidelines and permission of the national authorities (Permission No 0241). The PCV2 Cap-encoding DNA fragments were amplified using Phusion High-Fidelity DNA Polymerase (Thermo Fisher Scientific Baltics) to introduce BcuI sites at the 5′ end 3′ ends of the PCR fragment using primers CV-CP-Dir 5′CGACTAGTATGACGTATCCAAGGAGGCGTTAC CG and CV-CP-rev 5′CGACTAGTTAGGG GTTAAGTGGGGGGTCTTTAAG. Cloned gene variants were sequenced. Additionally, the gene encoding PCV2b-Cap protein 622 which appeared most common in Lithuanian pigs samples was codon optimized for expression in yeast (GenBank KJ128275). The gene of PCV2b Cap 622 with yeast-optimized codons (622S) was synthesized by GenScript. All four PCV2 Cap protein-encoding genes were cut with BcuI and subcloned into the XbaI site of the yeast expression vector pFX7 for target protein expression [28].

In addition, the gene encoding PCV1 Cap protein was synthesized by GenScript and subcloned into the XbaI site of the yeast expression vector pFX7.

### Detection of PCV2 virus DNA by PCR

Two-hundred microliters of porcine serum samples were used to isolate viral genomic DNA with GeneJET™ Viral RNA/DNA Purification Kit (Thermo Fisher Scientific Baltics) according to the manufacturer’s recommendations. Three pairs of primers (Rep-Bdir 5′ gccacctgggtgtggtaaaagcaaatg and PCV2-Xdir 5′tctagatgataactttgtaacaaaggc; Rep-Bdir 5′ gccacctgggtgtggtaaaagcaaatg and PCV-cap-Csq 5′ccgtgtaaccatgtatgtacaattcag; Rep-Brev 5′gtggccccacaatgacgtgtacattag and PCV2-revsq cggtaacgcctccttggatacgtc) were used to amplify PCV2 viral DNA fragments from isolated DNA.

### Yeast strains, growth media and cultivation conditions

The constructed plasmids pFX7-PCV2Cap-521, pFX7-PCV2Cap-113, pFX7-PCV2Cap-622, pFX7-PCV2Cap-622S and pFX7 were used for the transformation of *S. cerevisiae* strain AH22-214 (*a, leu2-3,112, his4-519*). Yeast transformants harboring plasmids with PCV2-cap encoding genes were grown in YEPD medium (yeast extract 1%, peptone 2%, and glucose 2%) supplemented with 5 mM formaldehyde overnight at 30°C and recombinant protein expression was induced after transferring yeast cells into induction medium YEPG (yeast extract 1%, peptone 2%, and galactose 3%) supplemented with 5 mM formaldehyde and culturing for additional 18 h. Yeast biomass harboring recombinant proteins was harvested by centrifugation and was stored at −20°C until use. Yeast transformed with vector pFX7 without any insert was used as a negative control.

### SDS-PAGE and western blotting

Proteins were analyzed by electrophoresis on 12% sodium dodecylsulfate-polyacrylamide gels (SDS-PAGE) followed by Coomassie brilliant blue staining. Briefly, 20–50 mg of yeast cell pellets were collected by centrifugation, washed with distillate water and suspended in 20–50 μL of DB 150 buffer (150 mM NaCl, 1 mM CaCl_2_, 0.001% Trition X-100, 0.25 M L-Arginine 10% glycerol in 10 mM Tris/HCl-buffer, pH 6.5). An equal volume of glass beads was added and the cells were lysed by vortexing for 7 min with cooling on ice for 1 min between each vortexing step. The samples of whole yeast lysates, the supernatant obtained after centrifugation of whole yeast lysate and the purified proteins were analyzed by SDS-PAGE. For this purpose, the samples were mixed with the SDS-PAGE sample buffer (Thermo Fisher Scientific Baltics), boiled for 5 min, applied to a SDS-PAGE and run in SDS-Tris-glycine buffer. Protein bands in SDS-PAGE were visualized by staining with Coomassie brilliant blue (Sigma–Aldrich, St. Louis, MO, USA) or electro-transferred to Immobilon P membrane (Millipore, Bedford, MA, USA). The membranes were blocked with 5% milk in TTBS (0.1% Tween 20, 0.1 M Tris, 0.3 M NaCl, pH 7.4) and incubated overnight in the relevant antibody solutions at room temperature (RT). Porcine serum and the MAbs were diluted 1: 600 and 1:1,000, respectively, in TBS with 0.1% Tween 20. After incubation with the diluted antibodies, the membranes were incubated for 2 hours with HRP-labeled anti-mouse IgG conjugate (Bio-Rad, Hercules, CA, USA) diluted 1:5000 or rabbit anti-pig IgG (Fab’2) conjugate (LifeSpan Biosciences, Seattle, USA) diluted 1:10000. The peroxidase-mediated staining was performed by adding 4-chloro-1-naphtol and H_2_O_2_ (Fluka, Buchs, Switzerland).

### Purification and characterization of PCV2-Cap VLPs

After centrifugation of yeast lysate, the supernatant containing PCV2-Cap proteins was collected and loaded onto a 20–69% sucrose gradient. Subsequently, a centrifugation at 100,000 × g (Beckman Optima LE-80 K Ultracentrifuge, Brea, CA, USA) overnight at 4°C followed. Thereafter, fractions of 3 mL were collected and samples were subjected to SDS-PAGE followed by Coomassie brilliant blue staining. Fractions showing a protein band with an apparent molecular mass of approximately 27 kDa corresponding to PCV2 Cap protein were pooled and diluted in DB150 buffer. The mixture was subjected to the ultracentrifugation on CsCl gradient with densities from 1.23 to 1.46 g/mL overnight at 100,000 × g (Beckman). Collected fractions were analyzed as described above. After pooling of the Cap-containing fractions, recombinant PCV2-Cap VLPs were diluted and precipitated by ultracentrifugation for 4 h at 100,000 × g (Beckman). The pellets were dissolved in PBS and dialyzed against PBS overnight. The dialyzed PCV2-Cap VLPs were stored in PBS in 50% glycerol at −20°C until use.

The samples of purified recombinant PCV2-Cap proteins were placed on 300-mesh carbon coated palladium grids, negatively stained with 2% aqueous uranyl acetate solution and examined by Morgagni 268 electron microscope (FEI Inc., Hillsboro, OR, USA).

### Immunization of mice and generation of monoclonal antibodies

All procedures involving laboratory mice were performed at the breeding colony of the Center for Innovative Medicine (Vilnius, Lithuania) under controlled laboratory conditions in strict accordance with Lithuanian and European legislation.

To generate MAbs against the recombinant PCV2-Cap protein, three BALB/c mice were injected subcutaneously 3 times with 50 μg of recombinant PCV2-Cap VLPs at 21-day intervals. For a primary immunization, the antigen was emulsified in Complete Freund’s adjuvant (Sigma-Aldrich Co), the subsequent immunizations were performed with PCV2 Cap VLPs dissolved in PBS without adjuvant. After the 3rd immunization, serum specimens were collected to determine the titers of specific antibodies. Finally, the mouse with the highest anti-PCV2-Cap titer was boosted with the same dose of PCV2-Cap VLPs in PBS and sacrificed for hybridoma development.

Hybridomas were generated essentially as described previously [40]. Four days after the booster immunization, spleen cells of the immunized mouse were fused with mouse myeloma Sp2/0 cells using PEG 1500 as a fusion agent (PEG/DMSO solution, HybriMax, Sigma-Aldrich Co). Hybrid cells were selected in growth medium supplemented with hypoxanthine, aminopterin and thymidine (50 × HAT media supplement, Sigma-Aldrich Co). Viable clones were screened by an indirect ELISA using 96-well microtiter plates coated with PCV2-Cap VLPs. Positive clones were stabilized by limiting dilution cloning on macrophage feeder layer. Hybridoma cells were maintained in complete Dulbecco’s modified Eagle’s medium (DMEM) containing 15% fetal calf serum (FCS, Biochrom, Cambridge, UK) and antibiotics. Heavy chain types of MAbs were determined by ELISA using Monoclonal Antibody Isotyping Kit (ISO-2, Sigma-Aldrich Co).

### Indirect immunofluorescence assay

Indirect immunofluorescence assay (IFA) was performed using commercial IFA slides for detection of PCV2-infected (virus strain T-657) swine testicle cells (PT-1) and PCV1-infected PK cells using commercially available slides (VMRD, Inc, Pullman, WA, USA) as recommended by the manufacturer. Briefly, 50 μL of cell culture supernatant of hybridomas #14G10, #24C7 and #7C4 was added to the slides in a dilution of 1:50 in PBS with 1% BSA or undiluted. The negative control MAb #4 F11 directed against parainfluenza type 3 N protein [41] was used at the same dilution. Detection was done by incubation with a fluorescein isothiocyanate (FITC)-conjugated anti-mouse immunoglobulin (H + L) goat IgG (DakoCytomation) diluted 1:20 in PBS. Cells were counter-stained with Evans Blue and IFA slides were analyzed using Olympus IX-70 fluorescence microscope (Olympus, Tokyo, Japan) with 40× objective at an excitation of 495 nm and Image-pro Plus Version 7.0.

### Serum samples

The porcine sera used in the study consisted of a collection of 409 samples obtained from 4–7 month old pigs from different regions in Lithuania. Samples were stored at −70°C prior to testing.

### Indirect ELISA

The specificity of mouse antisera, MAbs and swine sera was tested by an indirect ELISA. For testing the mouse antisera and the MAbs microtiter plates (Nunc MaxiSorp, Roskilde, Denmark) were coated with the respective antigens by adding 100 μL of the antigen solution (2 μg/mL) in coating buffer (50 mM sodium carbonate, pH 9.5) and incubation overnight at +4°C. The plates were blocked for 30 min at RT with 1% bovine serum albumin (BSA) in PBS and then incubated with either undiluted hybridoma supernatant or serially diluted MAbs for 1 h at RT.

For the indirect IgG PCV2-Cap VLP ELISA microtiter plates (Nerbe plus, Winsen/Luhe, Germany) were coated with 100 μL per well of recombinant PCV2 Cap VLPs (variant 622, GenBank: AHN49776) diluted in coating buffer to the concentration of 0.5 μg/mL and incubated overnight at +4°C. This concentration was determined by titration to reach optimal conditions for sensitivity and specificity. Control antigen - hamster polyomavirus (HaPyV) VP1 protein VLPs [28] was diluted to the concentration of 2 μg/mL. The coated plates were blocked with 150 μL/well of Roti-block (Carl Roth, Karlsruhe, Germany) for 1 h at room temperature. Plates were rinsed three times with washing buffer (PBS containing 0.1% Tween-20). The optimal serum dilution was determined by checkerboard titration (1:100, 1:200, 1:400, 1:800, 1:1600, 1:3200) in serum diluent (PBS containing 1% BSA and 0.05% Tween-20).

After 1 h of incubation the plates were rinsed five times with washing buffer (PBS containing 0.1% Tween-20) and incubated for 1 h at RT with HRP-labeled anti-mouse IgG (Bio-Rad, diluted 1:5000 in PBS-T) or rabbit anti-pig IgG (Fab’2; LifeSpan Biosciences, Seattle, USA) diluted 1:15 000 in PBS, containing 1% BSA and 0.05% Tween-20. The plates were washed as described above. Binding of specific antibodies was detected by the addition of 100 μL/well of TMB ready-to-use substrate (Sigma) or TMB substrate (Clinical Science Products, Mansfield, USA). After 10 min of incubation the reaction was stopped by adding 100 μL/well of 10% sulphuric acid and the optical density (OD) was measured at 450 nm (reference filter 620 nm) in a microtiter plate reader (Tecan, Groedig, Austria).

### Characterization of serum samples by the commercial test

Serum samples were assayed for the presence of anti-PCV2 antibodies using a commercial SERELISA PCV2 Ab Mono Blocking Kit (Synbiotics, Lyon, France) as recommended by the manufacturer. This ELISA is an epitope-blocking assay in which labeled MAb against PCV2 Cap protein is blocked from binding to the PCV2-coated microtiter plate if the serum sample contains antibody against PCV2. The test was performed according to the recommendations of the manufacturer. Data management and calculations were performed using software provided by the manufacturer. Any sample presenting a S/N ratio ≤ to 0.4 and a titer >350 is considered positive for the presence of PCV2 Cap –specific antibodies in pig serum. Any sample presenting a S/N ratio >0.4 and the titer ≤350 is considered as “low antibody in serum” or non-reactive.

## Abbreviations

PCV2: Porcine circovirus type 2
Cap: Capsid protein
VLPs: Virus-like particles
MAbs: Monoclonal antibodies
PCVAD: PCV2-associated diseases
PMWS: Postweaning multisystemic wasting syndrome
PDNS: Nephropathy syndrome
DCs: Dendritic cells
HaPyV: Hamster polyomavirus
IFA: Immunofluorescence assay
QIFA: Quantitative immunofluorescence assay
DMEM: Dulbecco’s modified Eagle’s medium
